# M1 macrophage infiltration exacerbate muscle/bone atrophy after peripheral nerve injury

**DOI:** 10.1186/s12891-020-3069-z

**Published:** 2020-01-20

**Authors:** Nobuhiro Shimada, Asuka Sakata, Takashi Igarashi, Mamoru Takeuchi, Satoshi Nishimura

**Affiliations:** 1 Center for Molecular Medicine, Jichi Medical University, 3311-1, Yakushiji, Shimotsuke, Tochigi, 329-0498 Japan; 2 Department of Anesthesiology and Critical Care Medicine, Jichi Medical University, 3311-1, Yakushiji, Shimotsuke, Tochigi, 329-0498 Japan; 3 Department of Pediatrics, Nara Medical University, 840, Shijocho, Kashihara, Nara, 634-0813 Japan

**Keywords:** M1 macrophage, Peripheral nerve injury, Muscle atrophy, Bone atrophy

## Abstract

**Background:**

Peripheral nerve injury causes limb muscle/bone atrophy, leading to chronic pain. However, the mechanisms underlying muscle/bone atrophy after peripheral nerve injury remain unknown. It was recently reported that M1 macrophages are the main factors responsible for neurogenic inflammation after peripheral nerve injury. We hypothesized that M1 macrophages are important in muscle/bone atrophy after nerve injury. Therefore, we investigated the influence of M1 macrophages on muscle/bone atrophy after nerve injury in mice to prevent muscle/bone atrophy by suppressing M1 macrophages.

**Methods:**

Hindlimb muscle weight and total bone density were measured in a chronic constriction injury (CCI) mouse model. Immunohistochemical analysis and intravital microscopy were performed to visualize hindlimb muscles/bones, and cells were quantified using flow cytometry. We compared M1 macrophage infiltration into muscles/bones and muscle/bone atrophy between macrophage depletion and untreated groups. We also investigated muscle/bone atrophy using administration models for anti-inflammatory and neuropathic pain drugs.

**Results:**

Peripheral nerve injury caused significant reduction in muscle weight and total bone density at 1 and 3 weeks after CCI, respectively, compared with that in controls. Osteoclast numbers were significantly higher at 1 week after CCI in the CCI group than in the control group. M1 macrophage infiltration into muscles was observed from 2 h after CCI via intravital microscopy and 1 week after CCI, and it was significantly higher 1 week after CCI than in the control group. In the macrophage depletion group, dexamethasone, pregabalin, and loxoprofen groups, M1 macrophage infiltration into muscles/bones was significantly lower and muscle weight and total bone density were significantly higher than in the untreated group.

**Conclusions:**

M1 macrophage infiltration exacerbates muscle/bone atrophy after peripheral nerve injury. By suppressing M1 macrophages at the neural injury local site, muscle/bone atrophy could be avoided.

## Background

Peripheral nerve injury causes limb muscle/bone atrophy [1], which worsens patients’ functional prognosis and leads to chronic pain [2–4]. Such atrophy is related to pain behaviors and immobilization [2, 3]. However, some studies have reported that muscle/bone atrophy after neural injury is difficult to explain only based on immobilization due to pain [5, 6]; many cases of muscle/bone atrophy are resistant to rehabilitation [7, 8]. The pathological significance of muscle/bone atrophy after peripheral nerve injury remains unknown.

Local inflammation associated with peripheral nerve injury (called neurogenic inflammation) exacerbates pain and involves chronic pain [9–11]. In neurogenic inflammation, after nerve injury, macrophage, neutrophil, and lymphocyte accumulation in the peripheral nervous system contributes to peripheral sensitization, which is mediated by several cytokines such as tumor necrosis factor-α (TNFα), interleukin-1β (IL-1β), chemokine (C–C motif) ligand 2 (CCL2), and C–C chemokine receptor 2 (CCR2). M1 macrophages are reportedly important in neurogenic inflammation and amplify pain [12–17].

We hypothesized that M1 macrophages are important for muscle/bone atrophy after nerve injury such as pain accompanied by neurogenic inflammation. Muscle/bone atrophy after nerve injury can be avoided by suppressing macrophages. We clarified the influence of M1 macrophages on muscle/bone atrophy after nerve injury in mice. For this purpose, we evaluated the degree of muscle/bone atrophy in a neuropathic pain mouse model and examined local inflammatory cell and cytokine changes while focusing on M1 macrophages. We investigated whether M1 macrophage depletion by clodronate liposomes avoids muscle/bone atrophy and examined the effects of anti-inflammatory drugs and drugs used for neuropathic pain.

## Methods

### Study aim, design, and setting

#### Mice

**Fig. 1:**
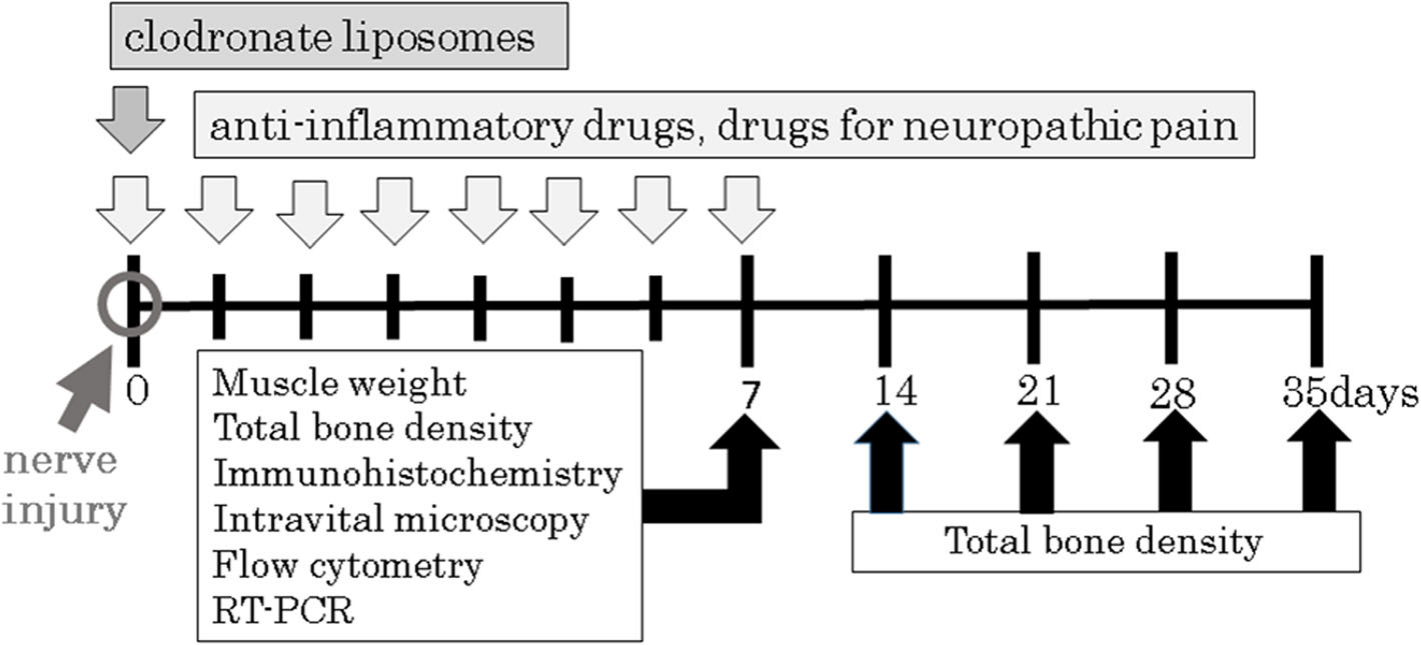
Experimental time course

This study adheres to the applicable Animal Research: Reporting In Vivo Experiments guidelines. The experiments were approved by the Institutional Animal Experiment Committee of Jichi Medical University (17217–01). Tables 1 and 2 provides information about the groups (total = 34), numbers of mice used, and treatment provided to each group. Figure 1 shows the experimental time course. In brief, 6–7-week-old male wild-type C57BL/6 J, C57BL/6-Tg (CAG-EGFP; SLC, Hamamatsu, Japan), and LysM Cre tandem TOMATO (CLEA, Tokyo, Japan) mice (19–23 g) were maintained in individual cages with a 12-h light/dark cycle at a constant temperature and provided ad libitum access to water and food. No mice had any abnormal health condition before the experiments. Invasive procedures were performed under general anesthesia.

**Table 1:** Group allocation and testing for C57BL/6 J mice

C57BL/6 J Mice	Control	CCI Surgery						
		No Tx	Clod	Dex	Preg	Loxo	Neuro	Amitryp
Muscle weight	8	8	8	8	8	8	–	–
Total bone density	8	8	8	8	8	8	–	–
Immunohistochemical analysis	8	8	8	8	8	8	8	8
Flow cytometry	8	8	8	8	8	8	8	8
RT-PCR	8	8	–	–	–	–	–	–

A total of 30 × 8 = 240 mice were tested

Control, surgery without chronic constriction injury (CCI); No Tx, no treatment; Clod, Clodronate liposome; Dex, Dexamethasone; Preg, Pregabalin; Loxo, Loxoprofen; Neuro, Neurotropin; Amitryp, Amitriptyline

**Table 2:** Intravital microscopy group allocation and testing

Test/Drug	Control	CCI-No Tx
C57BL/6-Tg (CAG-EGFP)	8	8
LysM Cre tandem TOMATO	8	8

A total of 4 × 8 = 32 mice were tested. C57BL/6-Tg (CAG-EGFP) mice were tested immediately after chronic constriction injury (CCI), whereas LysM Cre tandem TOMATO mice were tested 1 week after CCI

No Tx = no treatment

#### Animal models

Chronic constriction injury (CCI) was induced as described previously [18]. Briefly, mice were anesthetized with 1.5% isoflurane; the right common sciatic nerve was exposed by blunt dissection through the biceps femoris muscle. Four ligatures (5–0 Monocryl monofilament), positioned 1 mm apart, were tied loosely around the nerve. The muscle and skin layers were closed using 5–0 silk thread. In control mice, the right common sciatic nerve was exposed, but not ligated.

To investigate the influence of macrophages on muscle/bone atrophy after CCI, they were depleted by a single injection of clodronate liposomes (Xigieia Bioscience, Tokyo, Japan) into the tail veins (100 μg/mouse) immediately after CCI.

We also tried to prevent muscle/bone atrophy due to CCI using anti-inflammatory and neuropathic pain drugs. Immediately after CCI, dexamethasone (2 mg/kg subcutaneously; Fuji Pharma Co., Ltd., Toyama, Japan), pregabalin (30 mg/kg orally; Pfizer Japan, Inc., Tokyo, Japan), loxoprofen (3 mg/kg orally; Daiichi Sankyo, Ltd., Tokyo, Japan), neurotropin (60 NU/kg intraperitoneally; Nippon Zoki Pharmaceutical Co., Ltd., Osaka, Japan), and amitriptyline (10 mg/kg orally; Nichi-Iko Pharmaceutical Co., Ltd., Toyama, Japan) were administered daily for 1 week. Mice were randomly assigned to each group.

#### Muscle weight measurement

To evaluate muscle atrophy, 1 week after CCI, C57BL/6 J mice were sacrificed via cervical dislocation. The right biceps femoris and gastrocnemius muscles were collected; muscle weights were measured.

#### Total bone density

To evaluate bone atrophy, total hindlimb bone density was measured by computed tomography (CT) in C57BL/6 J mice anesthetized with 1.5% isoflurane (Latheta Laboratory CT; Hitachi, Tokyo, Japan). CCI was induced, and CT of the hindlimbs was performed every week for 5 weeks. Images were analyzed using CT scanner software. Total bone density of the femur and tibia were measured using 0.5-mm slices; their average values were compared.

#### Immunohistochemical analysis

Inflammatory cells and osteoclasts in the femur were analyzed by immunohistochemical analysis. 1 week after CCI, C57BL/6 J mice were sacrificed via cervical dislocation, and right femurs were collected. Pathological specimen preparation and immunostaining [tartrate-resistant acid phosphatase (TRAP) staining] were outsourced (Kyodo Byori, Kobe, Japan). Images were captured on a FSX100 microscope (Olympus, Tokyo, Japan) using a × 4 (N.A. 0.1) objective and analyzed with the Fiji win64 software (Rasband, W.S., ImageJ; National Institutes of Health, Bethesda, MD, USA). Under a 1.5- × 2-mm view, osteoclasts were separated by a single color according to the difference in staining intensity; osteoclast numbers were compared.

#### Intravital microscopy

To visualize inflammatory cell dynamics in the nerve and surrounding tissues, we used in vivo multiphoton microscopy [19]. 1 week after CCI, LysM Cre tandem TOMATO mice were anesthetized by urethane injection (1.5 g/kg); the hindlimb skin was removed. The hindlimb tissue was visualized under an inverted microscope (Eclipse Ti; Nikon). Further, fluorescein isothiocyanate–dextran (5 mg/mouse; Merck KGaA, Darmstadt, Germany) and Hoechst 33342 (3 mg/mouse; Thermo Fisher Scientific, Waltham, MA, USA) were injected into the tail vein to visualize cell dynamics and blood flow. The tissues were excited at a wavelength of 920 nm using a Ti:sapphire laser (Vision II; Coherent, Inc., Santa Clara, CA, USA), and images were captured as XY images using an A1R-MP system (Nikon). A × 40 (N.A. 1.15) water immersion objective lens (Nikon) was used. The pixel dwell time was set at 0.1 μs. To observe inflammatory response in the acute phase, CAG-EGFP mice were anesthetized immediately after CCI; Rhodamine B–dextran (5 mg/mouse; Thermo Fisher Scientific), Hoechst 33342 (3 mg/mouse), and F4/80 Alx647 (25 μg/mouse; BioLegend, San Diego, CA, USA) were injected into the tail vein. We continuously observed the sciatic nerve and surrounding tissues for 3 h after the injection. Collected data were analyzed via an automatic algorithm using the NIS-Elements and Fiji win64 software. Under a 500- × 500-μm view, macrophages were separated according to a single color based on the difference in fluorescence color, and their numbers were compared.

#### Flow Cytometry

Cell infiltration into muscles was analyzed by flow cytometry. 1 week after CCI, C57BL/6 J mice were sacrificed via cervical dislocation; the right biceps femoris and gastrocnemius muscles were collected, minced, and treated with collagenase. Cells were isolated, washed twice with phosphate-buffered saline, incubated for 8.5 min in erythrocyte lysis buffer, and finally suspended in phosphate-buffered saline. Next, isolated cells were incubated with an Fc Block antibody (BD Biosciences, Bedford, MA, USA) for 15 min on ice, labeled with dye-conjugated antibodies (CD45-FITC, 25 μg/mL; Ly6G-BV605, 10 μg/mL; F4/80-PE, 10 μg/mL; CD11b-BV711, 2.5 μg/mL; CD301-Alx647, 2.5 μg/mL; Ly6C-BV421, 2.5 μg/mL; BioLegend), and analyzed by flow cytometry using BD LSR Fortessa (BD Biosciences) and FlowJo V10 (Tomy Digital Biology, Tokyo, Japan). DRAQ7 (BioLegend) was used to exclude dead cells.

#### RNA isolation and reverse transcription polymerase chain reaction (RT-PCR)

To investigate cytokine changes in the muscles after CCI, RT-PCR was performed. 1 week after CCI, C57BL/6 J mice were sacrificed, and the right biceps femoris and gastrocnemius muscles were collected; RNA was extracted from the muscle tissues using Trizol (Thermo Fisher Scientific). cDNA was synthesized using PrimeScript reverse transcriptase (Takara Bio, Kusatsu, Japan). Taqman probes of TNFα, IL1-β, CCL2, and CCR2 (Thermo Fisher Scientific) were used in RT-PCR, and it was performed with 10 ng cDNA using a Vii A real-time PCR system (Thermo Fisher Scientific). Processing of raw data and normalization of the relative quantities were performed using the ΔΔ-Ct-method. mRNA expression levels are expressed relative to the control group.

### Statistical analysis

Statistical analysis was performed using Graphpad Prism7 (GraphPad Software, Inc., San Diego, CA, USA). All data were of equal variance in the F test; however, in the D’Agostino–Pearson normality test, except RT-PCR data, all data were not normally distributed. Taking into account that *n* = 8 is the least number of samples detectable by the D’Agostino–Pearson normality test, a nonparametric test was chosen, except RT-PCR. Mann–Whitney *U*-test was used to compare the groups. The Kruskal–Wallis test was used to compare ≥3 groups. The Friedman test was used for repeated measurements of the same sample. Dunn’s multiple comparison test was used for post hoc tests. The results are expressed as median and interquartile range. Because RT-PCR data were normally distributed, Student’s *t*-test was selected as the parametric test. Results were expressed as means ± standard deviation (SD). *P*-values of < 0.05 were considered statistically significant.

Sample sizes were calculated using GPower 3.1 (Heinrich-Heine-University, Düsseldorf, Germany) for primary outcomes [muscle weight (control group vs. CCI group, 1 week after CCI) and total bone density (CCI group, 0–5 weeks after CCI)] based on pilot studies on 5 mice. To detect a 50 mg decrease in muscle weight based on an SD of 30 mg, *n* = 8 in each group was considered appropriate (α = 0.05; 1 − β = 0.8). To detect a 60 mg/cm^3^ decrease in total bone density based on an SD of 50 mg/cm^3^, n = 8 in each group was considered appropriate (α = 0.05; 1 − β = 0.8). Therefore, n = 8 was chosen for each group (total groups = 34, total *n* = 272).

## Results

### Muscle/bone atrophy occurs in the early phase after CCI

In neurogenic inflammation after nerve injury, M1 macrophage infiltration into peripheral nerves has been reported to be important in pain amplification [9–17]. We investigated the influence of M1 macrophages on muscle/bone atrophy after nerve injury in mice. The numerical values obtained in this study are shown in Additional file 1.

**Fig. 2:**
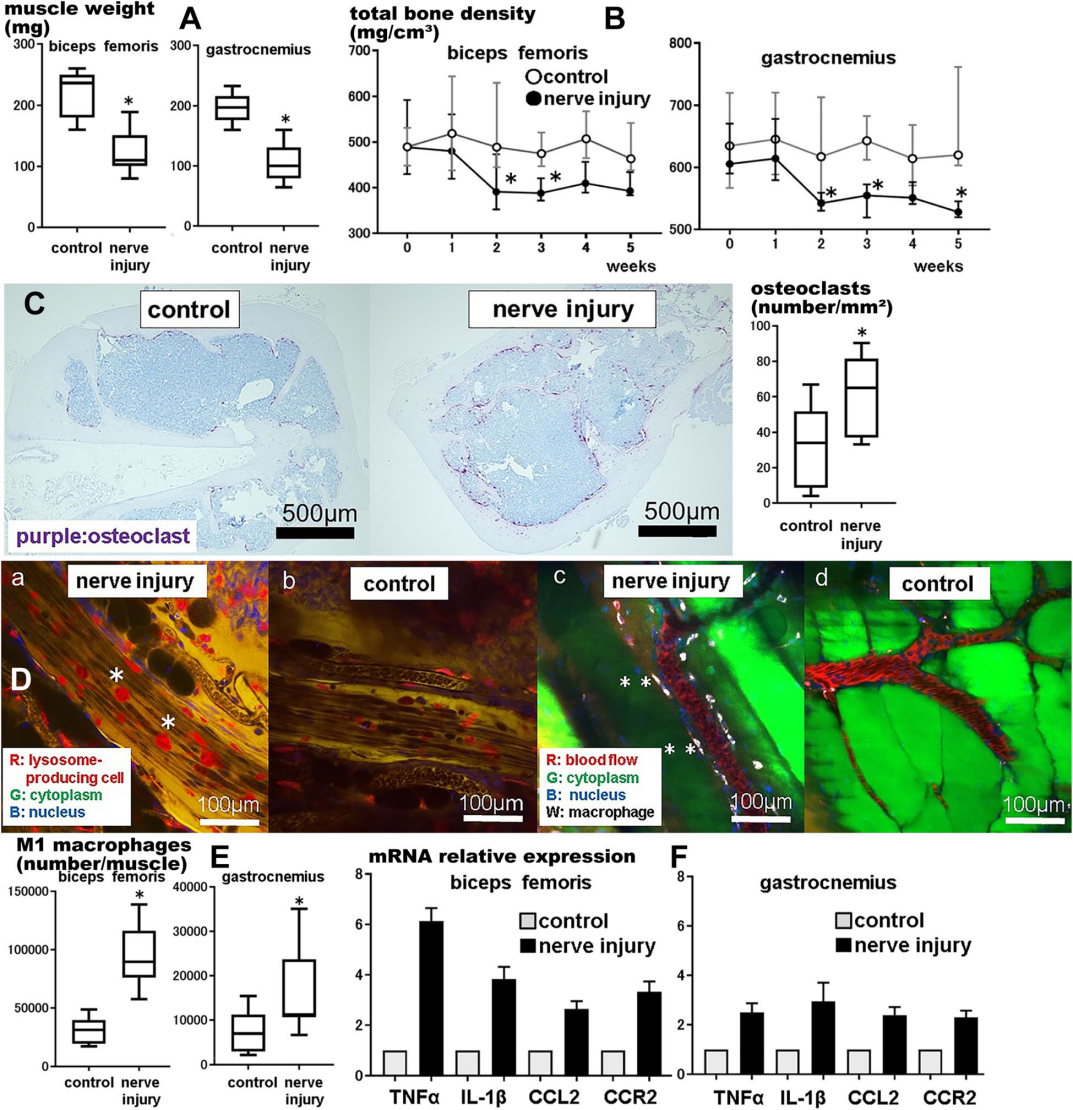
M1 macrophages’ influence on muscle/bone atrophy after nerve injury. We used a chronic constriction injury (CCI) mouse model as a nerve injury model. **a** Hindlimb muscle weight was lower in the nerve injury group 1 week postoperatively than in the control group (*n* = 8, *P < 0.05). *Center lines* of box plot indicate median, *box limits* indicate the 25th and 75th percentiles, and *whiskers* extend to minimum and maximum values. **b** Total hindlimb bone density decreased 2 weeks postoperatively in 5 weeks in the nerve injury group (*n* = 8, *P < 0.05). *Dot plots* indicate median, and error bars indicate the 25th and 75th percentiles. **c** Osteoclast numbers (*purple*) were higher as per immunohistochemical analysis in the femur in the nerve injury group 1 week postoperatively than in the control group (*n* = 8, *P < 0.05). **d** Lysosome-producing cells (*) increased 1 week postoperatively in the nerve injury group as per intravital imaging of nerves and muscles of LysM Cre tandem TOMATO mice (**d-a,** control group: **d-b**; R: lysosome-producing cell, G: cytoplasm, and B: nucleus). In the acute phase, macrophage (**) infiltration from the blood vessels to the muscles was observed from 2 h on intravital imaging of the nerves and muscles of CAG-EGFP mice (**d-c**, control group: **d-d**; R: blood flow, G: cytoplasm, B: nucleus, and W: macrophage). **e** M1 macrophage numbers were higher as per flow cytometry of the muscles in the nerve injury group 1 week postoperatively than in the control group (*n* = 8, *P < 0.05). **f** Inflammatory cytokine numbers were higher as per reverse transcription polymerase chain reaction of the muscles in the nerve injury group 1 week postoperatively than in the control group (*n* = 8, *P < 0.05). The means for each group are shown with the *top line* of the bar. Error bars indicate standard deviation

One week after CCI in C57BL/6 J mice, biceps femoris and gastrocnemius muscle weights were significantly lower in the CCI group than in the control group (*P* = 0.001 and *P* < 0.001, respectively; Fig. 2a).

Total hindlimb bone density was measured by CT performed weekly for 5 weeks (Fig. 2b). Total femoral and tibial bone densities decreased significantly at 5 weeks after CCI (*P* = 0.005 and *P* < 0.001, respectively). Multiple comparisons with 0 weeks revealed that total femoral bone density decreased significantly at 2 and 3 weeks after CCI (*P* = 0.037 and *P* = 0.035, respectively); total tibial bone density decreased significantly at 2, 3, and 5 weeks after CCI (*P* = 0.025, *P* = 0.016, and *P* < 0.001, respectively). However, the control group showed no significant difference in total femoral and tibial bone densities at 5 weeks after CCI (*P* = 0.808 and *P* = 0.480, respectively).

### M1 macrophages infiltrate muscles/bones after CCI

We investigated the molecular mechanism underlying muscle/bone atrophy after CCI. We focused on neurogenic inflammation involvement, particularly of M1 macrophages, which has recently attracted attention because of neuropathic pain [12–17].

Inflammatory cells and osteoclasts in the femur were analyzed by immunohistochemical analysis. One week after CCI in C57BL/6 J mice, TRAP staining of the right femur revealed that osteoclast numbers (Fig. 2c) were significantly higher in the CCI group than in the control group (*P* = 0.043).

The nerve/muscle cell dynamics in LysM Cre tandem TOMATO mice were observed with intravital microscopy 1 week after CCI. Infiltration into lysosome-producing cells around the sciatic nerves was observed in the CCI group (Fig. 2 da); such infiltration was mild in the control group (Fig. 2db). To observe inflammatory responses in the acute phase, the hindlimbs of CAG-EGFP mice were observed immediately after CCI. In the CCI group, macrophage infiltration from blood vessels to muscles was observed 2 h after CCI (Fig. 2dc); this was not observed in the control group (Fig. 2dd).

M1 macrophage infiltration into the hindlimbs observed by intravital microscopy was quantified by flow cytometry performed 1 week after CCI (Fig. 2e). In the biceps femoris and gastrocnemius muscles, M1 macrophage numbers (Ly6C+/F4/80+ and CD301−/CD11b+) were significantly higher in the CCI group than in the control group (*P* < 0.001 and *P* = 0.049, respectively).

### TNFα, IL-1β, CCL2, and CCR2 are involved in M1 macrophage infiltration into muscles/bones after CCI

In neuropathic pain, inflammatory cytokines are involved in neurogenic inflammation [9–11]. We thought that inflammatory cytokines were also involved in M1 macrophage infiltration and muscle/bone atrophy after CCI. To investigate cytokine changes in biceps femoris and gastrocnemius muscles after CCI, RT-PCR was performed 1 week after CCI on C57BL/6 J mice. mRNA expression levels are expressed relative to those of the control group (Fig. 2f). Concerning the biceps femoris and gastrocnemius muscles, relative mRNA expression was significantly higher in the CCI group than in the control group for TNFα, IL-1β, CCL2, and CCR2 (*P* < 0.001 for all).

### M1 macrophage infiltration into muscles/bones is suppressed by clodronate liposomes

The second aim of this study was to clarify whether muscle/bone atrophy can be avoided by suppressing M1 macrophages. We depleted M1 macrophages using clodronate liposomes after CCI.

**Fig. 3:**
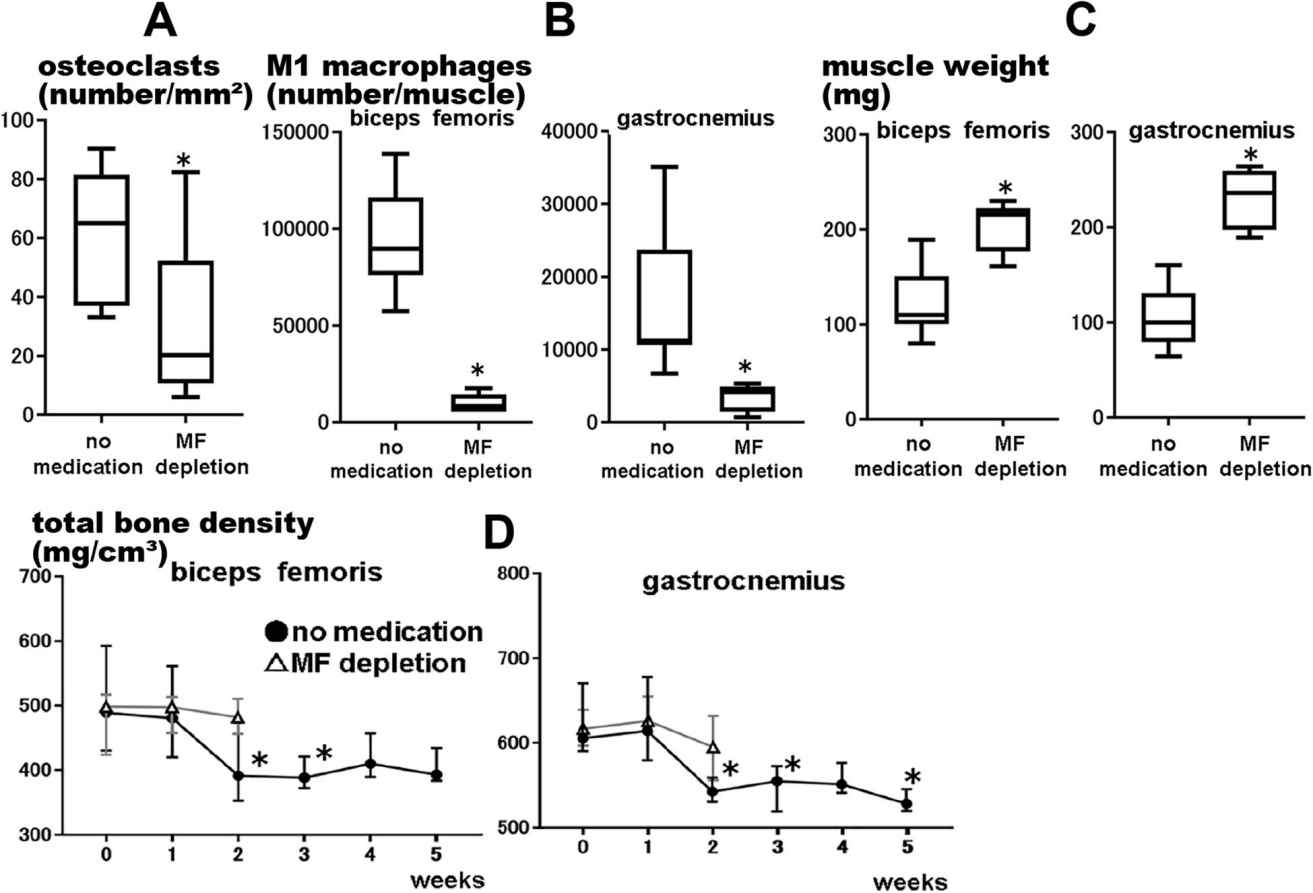
Reduced M1 macrophage numbers suppress M1 macrophage infiltration and muscle/bone atrophy after nerve injury. We used a chronic constriction injury (CCI) model mouse model as a nerve injury model. **a** Osteoclast numbers were lower as per immunohistochemical analysis of the femoral bone in the macrophage depletion group 1 week postoperatively than in the untreated group (*n* = 8, *P < 0.05). *Center lines* of *box plot* indicate median, *box limits* indicate the 25th and 75th percentiles, and *whiskers* extend to minimum and maximum values. **b** M1 macrophage numbers were lower as per flow cytometry of the muscles in the macrophage depletion group 1 week postoperatively than in the untreated group (*n* = 8, *P < 0.05). **c** Hindlimb muscle weight was higher in the macrophage depletion group1 week postoperatively than in the untreated group (*n* = 8, *P < 0.05). **d** In the macrophage depletion group, there was no significance of total femoral and tibial bone densities in 2 weeks after CCI (*n* = 8). *Dot plots* indicate median, and error bars indicate the 25th and 75th percentiles

One week after CCI, compared with the untreated group, the macrophage depletion group showed significantly lower numbers of osteoclasts on immunohistochemical analysis (*P* = 0.049; Fig. 3a) and of M1 macrophages on flow cytometry in the biceps femoris and gastrocnemius muscles (*P* < 0.001 and *P* < 0.001, respectively; Fig. 3b).

### M1 macrophage depletion suppressed muscle/bone atrophy after CCI

Muscle weight and total bone density changes were compared between the two groups to investigate their effects on muscle/bone atrophy.

In the macrophage depletion group, biceps femoris and gastrocnemius muscle weights were significantly higher 1 week after CCI than in the untreated group (*P* < 0.001 and *P* < 0.001, respectively; Fig. 3c), whereas there were no significant differences in total femoral and tibial bone densities at 2 weeks after CCI (*P* = 0.794 and *P* = 0.531, respectively; Fig. 3d).

### M1 macrophage infiltration into muscle/bone after nerve injury was significantly lower in dexamethasone, pregabalin, and loxoprofen groups than in the untreated group

To clarify whether M1 macrophage infiltration and muscle/bone atrophy can be avoided by clinically usable drugs, we used dexamethasone and loxoprofen as anti-inflammatory drugs (mainly for peripheral neuropathic pain) and pregabalin, neurotropin, and amitriptyline (the latter two for central pain) as neuropathic pain drugs.

**Fig. 4:**
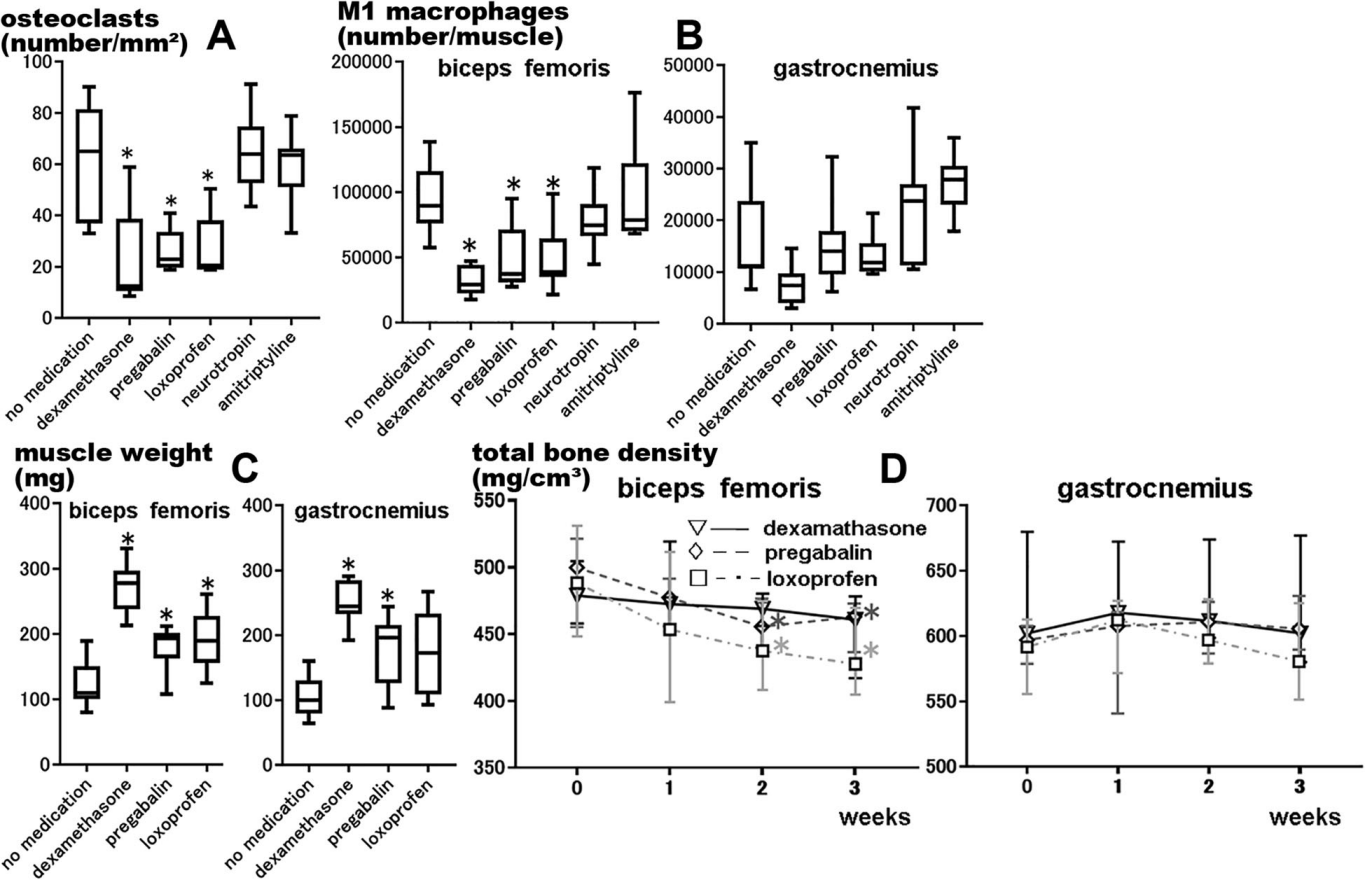
Dexamethasone, pregabalin, and loxoprofen suppress M1 macrophage infiltration and muscle/bone atrophy after nerve injury. We used a chronic constriction injury (CCI) model mouse model as a nerve injury model. **a** Osteoclast numbers were significantly lower as per immunohistochemical analysis of the femoral bone in drug administration groups (*n* = 8, *P < 0.05). *Center lines* of *box plot* indicate median, *box limits* indicate the 25th and 75th percentiles, and *whiskers* extend to minimum and maximum values. **b** The biceps femoris muscles 1 week after CCI showed that M1 macrophage numbers were significantly lower as per flow cytometry in the drug administration groups. In the gastrocnemius muscles 1 week after CCI, M1 macrophage numbers were significantly lower in the drug administration groups. (*n* = 8, *P < 0.05). **c** The weights of biceps femoris muscles 1 week after CCI were significantly higher in the drug administration groups. According to multiple comparisons to the untreated group, muscle weights were significantly higher in the dexamethasone, pregabalin, and loxoprofen groups. In the gastrocnemius muscles 1 week after CCI, muscle weights were significantly higher in the drug administration groups (*n* = 8, *P < 0.05). **d** Regarding the femur, there were no significant differences in total bone density in the dexamethasone group at 3 weeks after administration. Total bone density significantly decreased in the pregabalin and loxoprofen groups. Regarding the tibia, there were no significant differences in total bone density in all groups at 3 weeks after administration (*n* = 8, * *P* < 0.05). *Dot plots* indicate median and error bars indicate the 25th and 75th percentiles

One week after CCI, immunohistochemical analysis revealed that osteoclast numbers were significantly lower in the drug groups (*P* = 0.003). According to multiple comparisons with the untreated group, osteoclast numbers were significantly lower in the dexamethasone, pregabalin, and loxoprofen groups (*P* = 0.005, *P* = 0.038, and *P* = 0.042, respectively). There were no significant differences in osteoclast numbers between the neurotropin and amitriptyline groups and the untreated group (*P* > 0.99 and *P* > 0.99, respectively; Fig. 4a).

Flow cytometry of the biceps femoris muscles 1 week after CCI showed that M1 macrophage numbers were significantly lower in the drug administration groups (*P* = 0.002). On multiple comparisons with the untreated group, it was found that M1 macrophage numbers were significantly lower in the dexamethasone, pregabalin, and loxoprofen groups (*P* < 0.001, *P* = 0.043, and *P* = 0.034, respectively). There were no significant differences in M1 macrophage numbers between the neurotropin and amitriptyline groups and the untreated group (*P* > 0.99 and *P* > 0.99, respectively). In the gastrocnemius muscles 1 week after CCI, M1 macrophage numbers were significantly lower in the drug administration groups (*P* = 0.033). According to multiple comparisons with the untreated group, there were no significant differences in M1 macrophage numbers in any group (dexamethasone, *P* = 0.134; pregabalin, *P* > 0.99; loxoprofen, *P* > 0.99; neurotropin, *P* > 0.99; and amitriptyline, *P* = 0.096; Fig. 4b).

### Muscle weight and total bone density after nerve injury were significantly higher in dexamethasone, pregabalin, and loxoprofen groups than in the untreated group

Muscle weight and total bone density changes in the drug administration groups were compared with those in the untreated group to investigate their effects on muscle/bone atrophy. These examinations were conducted only for the dexamethasone, pregabalin, and loxoprofen groups, which showed a significant difference in M1 macrophage infiltration into muscles/bones.

One week after CCI, muscle weight was significantly higher in the drug administration groups (biceps femoris, *P* = 0.001 and gastrocnemius, *P* = 0.002). According to multiple comparisons with the untreated group, biceps femoral muscle weights were significantly higher in the dexamethasone, pregabalin, and loxoprofen groups (*P* < 0.001, *P* = 0.036, and *P* = 0.030, respectively) and gastrocnemius muscle weights were significantly higher in the dexamethasone and pregabalin groups (*P* < 0.001 and *P* = 0.046, respectively). However, there were no significant differences with the loxoprofen group (*P* = 0.085; Fig. 4c).

Regarding the femur, there were no significant differences in total bone density in the dexamethasone group at 3 weeks after administration (*P* = 0.176). Total bone density significantly decreased in the pregabalin and loxoprofen groups [pregabalin, *P* = 0.002, multiple comparisons with 0 week, significant difference at 2 (*P* = 0.011) and 3 (*P* = 0.002) weeks and loxoprofen, *P* = 0.001, multiple comparisons with 0 week, significant difference at 2 (*P* = 0.006) and 3 (*P* < 0.001) weeks]. Regarding the tibia, there were no significant differences in total bone density in all groups at 3 weeks after drug administration (dexamethasone, *P* = 0.789; pregabalin, *P* = 0.648; loxoprofen, *P* = 0.092; Fig. 4d).

## Discussion

Our study revealed that M1 macrophage infiltration exacerbates muscle/bone atrophy after peripheral nerve injury. To date, muscle/bone atrophy after nerve injury was thought to be related to pain behaviors and immobilization [2, 3]. Some studies reported that muscle/bone atrophy after nerve injury is difficult to explain by pain immobilization alone [5, 6], but these mechanisms were unknown. We focused on M1 macrophages, which have recently attracted attention because of neuropathic pain accompanied by neurogenic inflammation [12–17]. However, these studies focused only on neuropathic pain.

In neurogenic inflammation, Wallerian degeneration of peripheral nerves by M1 macrophages invading surrounding blood vessels has been reported to cause hyperalgesia [12]. Inflammatory cytokines such as TNFα, IL-1β, CCL2, and CCR2 are responsible to induce M1 macrophages in injured nerves [9–11]. Their levels significantly increased in muscle tissues after CCI in our study. We also observed that M1 macrophages invaded outside surrounding blood vessels, not muscles and bone tissues, on intravital microscopy. From these results, as a mechanism by which cytokine-induced M1 macrophages disrupt muscles and bone tissues, we believe that the cause of this disruption is not direct phagocytosis but mediators such as cytokines. Inflammatory disorders such as chronic obstructive pulmonary disease, rheumatoid arthritis, and inflammatory myopathies have recently been reported to display skeletal muscle atrophy [20]. In these diseases, the levels of inflammatory cytokines such as TNFα, interferon-γ, IL-6, and IL-8 increase, and they are thought to lead to muscle atrophy by reducing peripheral insulin action while increasing reactive oxygen species level and ischemia [21–23]. Muscle atrophy mediated by M1 macrophages, as observed in our study, may also occur via the same mechanism. In our study, muscle weight was measured for 1 week and total bone density was measured for up to 5 weeks. In the macrophage depletion group, total bone density was measured for up to 2 weeks due to exacerbation of the general condition of mice. In the drug administration groups, total bone density was measured for up to 3 weeks; because we obtained clear results within 2–3 weeks, mice were sacrificed early to avoid prolonged pain. In the future, changes in muscle/bone atrophy after nerve injury should be examined over a longer term. Further, in this study, we did not perform pain experiments such as behavior test, because assessment of pain has not been established for animal models of muscle/bone atrophy. This study should be linked to pain studies, particularly chronic pain studies. In this study, we used C57BL/6 J mice. Although there is a disadvantage of low bone density and altered macrophage response, C57BL/6 J mice have the advantage of high Th1 reactivity and that the involvement of innate and cell-mediated immunity in macrophages can be clarified. C57BL/6 J mice are highly sensitive to pain, used widely in pain studies, and often used in macrophage studies as a neuropathic pain model [16]. Therefore, this study conducted using C57BL/6 J mice can be considered useful as a foundation for future pain studies.

Regarding the depletion of macrophages using clodronate liposomes, mice were sacrificed within 4 weeks because they were weakened by infection and embolism of the excised macrophage mass. On the other hand, in the pilot study, the number of macrophages infiltrating muscles and bones did not decrease with the administration of a smaller amount of clodronate liposomes. In recent years, pain with bone metabolism abnormality after nerve injury has been reported as neuropathic bone pain, mainly in patients with complex regional pain syndrome, and bisphosphonate reportedly is useful not only for bone pain but also for neuropathic pain [24]. The optimal dosage of bisphosphonate necessary to prevent muscle/bone atrophy is a subject for future research. We studied dexamethasone and loxoprofen as anti-inflammatory drugs and pregabalin, amitriptyline, and neurotropin as neuropathic pain drugs because they can be linked to clinical research. The reason for using neuropathic pain drugs in addition to anti-inflammatory drugs is that pregabalin has been reported to be effective not only for neuropathic pain but also for neurogenic inflammation [25]. In the dexamethasone, pregabalin, and loxoprofen groups, M1 macrophage infiltration into muscles/bones was significantly lower, whereas muscle weight and total bone density were significantly higher than in the untreated group. These drugs might suppress muscle/bone atrophy after nerve injury via M1 macrophage suppression. Even in clinical practice, the use of steroids, nonsteroidal anti-inflammatory drugs, and antiepileptic drugs from the early stages may be effective in avoiding muscle/bone atrophy and chronic pain. Based on our results, steroids may be most effective against these conditions, but in clinical situations, the effect of steroids on neuropathic pain is controversial in terms of effectiveness and side effects [26]. Clinical studies on the short-term use of steroids after neural injury are desired targeting not only neuropathic pain but also muscle/bone atrophy. On the other hand, central sensitization is reportedly involved in neurogenic inflammation [9–11]. However, contrary to our expectations, amitriptyline and neurotropin, which mainly act on the central nervous system, were not effective in suppressing M1 macrophage infiltration into muscles/bones. Although the relationship between central sensitization in the dorsal horn of the spinal cord and muscle/bone atrophy should be considered further in future research, based on this study, central nervous system involvement in muscle/bone atrophy was considered mild because of the ineffectiveness of the studied drugs, whose analgesic pathways are mediated mainly by the central nervous system. In the future, a study that will clarify the mechanism underlying the suppression of M1 macrophages with these drugs is desired. However, from this study alone, we cannot deny the possibility that mechanisms other than M1 macrophage suppression are involved in muscle/bone atrophy suppression. Our results alone cannot be applied to clinical practice. In future clinical research, in addition to examining the avoidance of muscle/bone atrophy using medicines, muscle/bone atrophy measurement to diagnose neuropathic pain and judge therapeutic effects is necessary. It is necessary to investigate the possibility of suppressing chronic pain by avoiding muscle/bone atrophy.

As a limitation of this study, it is undeniable that muscle/bone atrophy following CCI might be caused by environmental factors such as immobility due to pain. Before this study, muscle weight and total bone density were observed over time in mice with hip, knee, and ankle joints fixed with ultraviolet-curing resins. Therefore, during the course of 1–2 weeks, muscle/bone atrophy of the fixed lower limb did not occur. The progress of muscle/bone atrophy following nerve injury in animal models has not been reported. On the other hand, in lower limb immobilization models, gastrocnemius muscle weight reportedly decreased by 22% after 1 week of immobilization [27], and these changes are smaller than those found in our study with a CCI model. In clinical practice, in humans, muscle mass reduction is 0.5%/day [28] and bone loss is 3–6%/month [6, 29] after spinal injury and immobilization. It is understood that muscle/bone atrophy in our study was due to the direct influence of neural injury.

Sufficient sample numbers could not be set for all experiments. The pilot study conducted to determine the number of samples in this study was performed only on the main study outcomes (muscle weight and total bone density). A pilot study of all experiments will require a large number of animals. In the interests of animal protection, the number of samples calculated for the main endpoint was used for other experiments. We did not adjust the analysis for multiple outcomes and multiple comparisons of several drugs against untreated controls. Therefore, the risk for type I errors is high, and the results should be viewed as hypothesis generating rather than that providing conclusive evidence.

## Conclusions

M1 macrophage infiltration exacerbates muscle/bone atrophy after peripheral nerve injury. By suppressing M1 macrophages at the local neural injury site, it was possible to avoid muscle/bone atrophy.

## Supplementary information

**Additional file 1.** The numerical values of animal experiments.

## Data Availability

All data generated or analyzed during this study are included in this published article and its supplementary information files.

## Abbreviations

CCI: Chronic constriction injury
CCL2: Chemokine (C–C motif) ligand 2
CCR2: C-C chemokine receptor 2
CT: Computed tomography
IL-1β: Interleukin-1β
RT-PCR: Reverse transcription polymerase chain reaction
SD: Standard deviation
TNFα: Tumor necrosis factor-α
TRAP: Tartrate-resistant acid phosphatase
