# Supplementary material for: M1 macrophage infiltration exacerbate muscle/bone atrophy after peripheral nerve injury
Source: BMC Musculoskelet Disord. 2020 Jan 20;21:44. doi: 10.1186/s12891-020-3069-z (PMC6971979; doi:10.1186/s12891-020-3069-z)
Supplement: Supplementary file 1 — Additional file 1. The numerical values of animal experiments. [file 12891_2020_3069_MOESM1_ESM.pdf]

The numeric results of muscle weight measurement

Muscle Weight (mg)		Median	IQR	Range
Control	Biceps femoris	237	180–250	160–260
	Gastrocnemius	197	176–216	160–233
CCI	Biceps femoris	110	101–151	80–189
	Gastrocnemius	100	79–131	64–160
MF depletion	Biceps femoris	216	177–223	161–230
	Gastrocnemius	236	197–260	189–264
Dexamethasone	Biceps femoris	278	238–297	213–331
	Gastrocnemius	245	232–285	192–291
Pregabalin	Biceps femoris	193	162–202	108–212
	Gastrocnemius	196	126–216	88–244
Loxoprofen	Biceps femoris	190	156–228	125–261
	Gastrocnemius	173	109–233	93–267

IQR, inter quartile range; CCI, chronic constriction injury; MF, macrophage.

The numeric results of total bone density measurement (control, CCI, macrophage depletion)

Total bone density (mg/cm^3)			0w	1w	2w	3w	4w	5w
control	femur	median	489	518	489	475	507	463
		IQR	448–531	438–643	445–629	447–520	465–567	438–541
		range	325–765	350–756	312–667	431–611	350–612	399–672
	tibia	median	635	646	617	661	663	632
		IQR	567–720	589–721	547–713	535–727	529–718	516–702
		range	304–889	338–783	384–881	298–892	308–879	388–892
CCI	femur	median	489	481	391	388	410	393
		IQR	430–593	429–561	353–474	372–421	389–457	383–434
		range	350–644	370–637	264–503	355–473	358–479	359–479
	tibia	median	605	614	542	555	550	528
		IQR	590–670	579–678	530–559	519–572	541–576	520–545
		range	320–783	349–798	254–796	254–753	269–732	266–737
MF depletion	femur	median	499	497	482	-	-	-
		IQR	424–517	458–514	456–511	-	-	-
		range	309–647	287–632	355–644	-	-	-
	tibia	median	617	626	595	-	-	-
		IQR	597–639	613–655	556–632	-	-	-
		range	270–871	318–865	329–797	-	-	-

IQR: inter quartile range, CCI: chronic constriction injury, MF: macrophage

The numeric results of total bone density measurement (drug administration groups)

Total bone density (mg/cm^3)			0w	1w	2w	3w	4w	5w
Dexamethasone	femur	median	479	472	469	461	-	-
		IQR	458–504	452–519	456–480	417–478	-	-
		range	348–642	335–685	345–619	321–619	-	-
	tibia	median	602	618	612	602	-	-
		IQR	588–680	613–672	599–674	580–677	-	-
		range	309–832	293–822	275–825	232–827	-	-
Pregabalin	femur	median	500	478	456	463	-	-
		IQR	455–522	456–492	440–477	437–473	-	-
		range	332–676	313–662	317–628	332–476	-	-
	tibia	median	597	608	611	606	-	-
		IQR	579–601	541–618	587–626	590–631	-	-
		range	333–785	285–822	270–807	266–845	-	-
Loxoprofen	femur	median	488	453	437	428	-	-
		IQR	448–531	399–511	408–474	405–469	-	-
		range	329–670	301–666	282–611	295–612	-	-
	tibia	median	592	612	597	581	-	-
		IQR	556–613	572–627	579–628	511–625	-	-
		range	322–782	294–832	249–853	255–829	-	-

IQR: inter quartile range

The numeric results of immunohistochemistry of femur

Osteoclasts (number/mm^2)	median	IQR	range
control	34	15–44	4–67
CCI	65	37–82	33–90
MF depletion	20	11–52	6–82
Dexamethasone	13	11–39	9–59
Pregabalin	23	20–34	19–41
Loxoprofen	21	19–38	18–50
Neurotropin	64	53–75	43–91
Amitriptyline	64	51–66	33–79

IQR: inter quartile range, CCI: chronic constriction injury, MF: macrophage

The numeric results of flow cytometry

M1 macrophage ($\times 10^5$ number/muscle)		median	IQR	range
control	biceps femoris	3.1	2.0–4.0	1.7–4.9
	gastrocnemius	0.7	3.0–1.1	0.2–1.5
CCI	biceps femoris	8.9	7.6–11.6	5.8–13.9
	gastrocnemius	1.1	1.1–2.4	0.7–3.5
MF depletion	biceps femoris	0.8	0.5–1.4	0.5–1.8
	gastrocnemius	0.4	0.1–0.5	0.1–0.5
Dexamethasone	biceps femoris	2.9	2.2–4.4	1.8–4.7
	gastrocnemius	0.7	0.4–1.0	0.3–1.5
Pregabalin	biceps femoris	3.7	3.1–7.2	2.8–9.5
	gastrocnemius	1.4	1.0–1.8	0.6–3.2
Loxoprofen	biceps femoris	3.9	3.5–6.5	2.2–9.9
	gastrocnemius	1.2	1.0–1.6	1.0–2.1
Neurotropin	biceps femoris	7.5	6.7–9.1	4.5–11.9
	gastrocnemius	2.4	1.1–2.7	1.1–4.2
Amitriptyline	biceps femoris	7.9	7.0–12.2	6.8–17.6
	gastrocnemius	2.8	2.3–3.1	1.8–3.6

IQR: inter quartile range, CCI: chronic constriction injury, MF: macrophage

The numeric results of reverse transcription polymerase chain reaction

mRNA relative expression			TNF α	IL−1 β	CCL2	CCR2
CCI	Biceps femoris	mean	6.1	3.8	2.6	3.3
		SD	0.5	0.5	0.3	0.4
		range	5.4–6.9	3.0–4.3	2.3–3.2	2.6–3.8
	gastrocnemius	mean	2.5	2.9	2.4	2.3
		SD	0.3	0.8	0.3	0.3
		range	2.1–3.1	2.1–3.7	2.1–3.0	2.0–2.8

CCI: chronic constriction injury, TNFα: tumor necrosis factor-α,

IL-1β: interleukin-1β, CCL2: chemokine (C-C motif) ligand2

CCR2: C-C chemokine receptor type2
